# Molecular Pathology, Diagnostics and Therapeutics: A Story of Success in 2022

**DOI:** 10.3390/ijms24055063

**Published:** 2023-03-06

**Authors:** Stephen A. Bustin

**Affiliations:** Medical Technology Research Centre, Anglia Ruskin University, Chelmsford CM1 1SQ, UK; stephen.bustin@gmail.com

## 1. Introduction

Molecular pathology, diagnostics and therapeutics are three closely related topics of critical importance in medical research and clinical practice. Understanding the biology of diseases at the molecular level by identifying molecular and pathway alterations is important for several reasons:

1. It can facilitate earlier and more accurate diagnoses of diseases, which are key to initiating appropriate treatments at the right time as well as reducing healthcare costs.
2. It enables the development of new drugs and the design of more effective therapies, including the development of treatments that are tailored to the individual patient’s genetic makeup.
3. It is useful for disease prevention by identifying individuals at risk of developing certain conditions, thus encouraging early targeted interventions and lifestyle changes.

This field has transitioned into an extensive range of molecular and cell methodologies and techniques in the clinical arena that analyse an equally wide range of samples and diseases. A vast amount of complex data is generated and results in a taxing bioinformatic, bioanalytic and statistical workload. The molecular pathology, diagnostics and therapeutics section of the International Journal of Molecular Science (IJMS) aims to provide a go-to home for high quality, innovative and definitive publications in this critical area of health research.

## 2. Review of 2022

The significance of this field is reflected in the important contribution made by the molecular pathology, diagnostics and therapeutics section to the IJMS. This section includes contributions from basic research all the way to clinical and forensic applications and is especially open to studies that challenge existing concepts. It accounted for 14.4% of papers published in the journal during 2022, with the number of publications increasing to 2356 out of 4995 submissions (47%), resulting in 1.5 million downloads. Papers published in 2020/21 were cited nearly 15,000 times, with the most cited publications in the areas of cardiovascular diseases, cancer/tumour treatment, neurodegenerative diseases, endocrine diseases, and COVID-19-related research. Of these, 163 papers (6.9%) were cited more than five times and 20 of those more than 10 times. Consequently, papers published in this section have contributed to the steady rise of the journal’s impact factor, which currently stands at 6.208. This increase in quality and quantity is nurtured by the increased number of specialist editorial board and topical advisory panel members and is sustained by the many expert reviewers, whose comments and contributions are an essential part of this rise. Together this helps to ensure that the research presented in our section is of high quality, that methods are clearly described, results are fully reported and that the conclusions drawn are supported by the data. As a consequence, our section and the journal as a whole are respected by the scientific community and that the research is widely cited.

Further to this topic, I would like to remind potential authors of the importance of method and data transparency, as both are critical components for evaluating the significance of reported results and any conclusions. For example, quantitative PCR (qPCR) and reverse transcription (RT)-qPCR are widely used technologies in this field, not least evidenced by the huge number of recent publications relating to SARS-CoV-2. Yet as an editor it continues to surprise me how little technical detail is generally included with the first submisssions of manuscripts and how inadequately results are presented. It is as though many authors either regard this technology as so common and standardised that no detail is required, or they have no understanding of its complexity of the various components such as RNA quality, PCR efficiency, primer specificity or significance of fold-changes in biomarker expression levels. I would urge authors to consult an earlier editorial describing the essential criteria that should accompany any submission to *IJMS* [1]. The same requirement for transparency is obviously important for any technique used in a published paper.

The percentage of primary research articles has increased to 60.1% in 2022, up from 52.2% in 2021. This is probably a reflection of the increasing impact factor of the journal, which encourages more researchers to trust it with their latest and most important research results and, in turn, fuels a virtuous circle of increased quality resulting in further increases in the impact factor. Conversely, this increase has led to a decrease in the number of review articles (36.6% from 45.9% in 2021). Clearly there is a need to strike a balance, since review articles tend to attract more citations and increase the scholarly impact of the journal. Nevertheless, as an editor I find it promising and exciting that so many authors choose our section for the dissemination of their precious data.

There has been an increasing emphasis on Special Issues, to which publications are invited by guest editors and panel members, with a consistent review process ensuring high quality as well as topicality. In 2022 there were 547 Special Issues launches, compared with 368 in 2021. Indeed, the vast majority of papers in 2022 were published as parts of Special Issues. Nevertheless, the rise in the impact factor and the corresponding increase in our reputation saw an increasing number of regular submissions to the journal. I anticipate that this trend will continue for three reasons: (i) the IJMS impact factor is higher than that of competitor journals, (ii) the steady increase in its impact factor is not mirrored by those journals and (iii) their article processing charges are higher. The open access format combined with reasonable processing charges are two important reasons for the success of the journal. Papers can be accessed by anyone with an internet connection, regardless of their location or affiliation, thus can reach a much wider audience. Combined with a rapid processing workflow, this makes research published in our section available to the scientific community as well as the public sooner, making our contributions more topical and relevant to current news cycles. The increased visibility and accessibility also help articles being cited more frequently, so adding to their impact and influence.

It follows, then, that the logistics of manuscript handling should be as clear, smooth, and rapid as possible as every author likes their manuscript to be reviewed and processed in as short a time frame as possible. IJMS in general and this section in particular have clear policies and guidelines on how to prepare and submit manuscripts, for ethical behaviour, and for the peer review process. The molecular pathology, diagnostics, and therapeutics section of the IJMS performs well, with a first decision provided to authors 16 days after submission and the median processing time being 36 days. This is remarkably swift, given that each manuscript is subject to strict peer review and considering the 16% increase in submissions since 2020. Feedback surveys indicate that authors appreciate our fast publication times, and the editors will strive to maintain this benefit for the future, whilst obviously ensuring that the section maintains the high paper quality.

Amongst the top 15 countries publishing in this section, most continue to come from Europe (including Russia) at 41.5%, slightly down on the 45% recorded in 2021. Contributions from Asia (24.2% vs. 23.4%) and North America (13.7% vs. 14.8%) are roughly the same and the appearance of contributions from Australia (1.9%) is welcome. Within those groupings, there has been a sizeable increase in manuscripts submitted from China (+5.1%) and decrease from South Korea (−4.4%). An analysis of author origin in the pathology category of the Web of Science reveals that of the top three countries, only China has a similar number of submissions to our journal (10.9% vs. 8.6%). The USA (41.3% vs. 12% and the UK (6.2% vs. 2%) are under-represented. I also note that there are few submissions from India, which is home to 3.5% of submissions on the Web of Science. In contrast, the journal is rather popular with researchers from Japan (5.3% vs. 7.3%), Germany (4.8% vs. 6.6%) and, especially, Italy (4.1% vs. 13.6%). Clearly there is work to be done to encourage authors in countries such as India, South Africa and South America to consider *IJMS* as a destination for their research outputs. Author origin and online readership are closely aligned, suggesting a good cooperative relationship with the section. The readership extends way beyond the top contributor nations, though, with 26% of the online readership from countries with fewer than 2% views.

Editorial board and topical advisory panel members are composed of experts in molecular pathology, diagnostics, therapeutics, and related field. Their understanding of, and indeed personal contribution to current research developments are important safeguards that allow unbiased and professional evaluation of the quality of the manuscripts submitted to the section. It is also important to note that the name of the academic editor accepting a manuscript after full peer review becomes associated with the published paper. This enhances the rigorous and unprejudiced review procedure, promotes maximum transparency for authors and readers alike, and provides a measure of responsibility if subsequent uncertainties arise.

Board and advisory panel members play an essential role in ensuring that published manuscripts are of the highest quality, report innovative research results and are published with transparent methods and appropriate statistical analyses. They are from a wide range of countries, with most concentrated in two countries, Italy and the USA. Clearly one aim for next year must be to recruit more academic editors from a wider range of countries, especially China.

## 3. Outlook for 2023

With awareness of the current pandemic fading, it is essential to maintain the spotlight on the importance of molecular pathology and diagnostics as critical components of any country’s public health infrastructure and their role in opening new therapeutic pathways. Hence, the focus in 2023 will be to make our section an even better platform for authors and guest editors of Special Issues to publish relevant, influential, and conclusive research results. Ultimately, the basis of journal’s reputation is the quality of its primary research papers; it is enhanced by the publication of authoritative reviews that become influential in shaping the debate surrounding public health. Our aim must be to continue to provide a first-class publication experience to authors and encourage the submission of leading-edge research papers as well as of forward-looking and open-minded profiles of current work and future goals.

## References

[1] Bustin S. (2013). Transparency of reporting in molecular diagnostics. Int. J. Mol. Sci..

